# High‐Starch Diet Prepartum Enhances IgG Concentration in Goat Colostrum Without Affecting the Transfer of Passive Immunity

**DOI:** 10.1111/jpn.70089

**Published:** 2026-06-30

**Authors:** Marta González‐Cabrera, Antonio Morales‐delaNuez, Anastasio Argüello, Alexandr Torres, Noemí Castro, Lorenzo Enrique Hernández‐Castellano

**Affiliations:** ^1^ IUSA‐ONEHEALTH 4 Animal Production and Biotechnology Group, Institute of Animal Health and Food Safety Universidad de Las Palmas de Gran Canaria, Campus Montaña Cardones Las Palmas de Gran Canaria Spain; ^2^ Unit of Animal Production, Pasture, and Forage in Arid and Subtropical Areas Canary Islands Institute for Agricultural Research San Cristóbal de La Laguna Spain

**Keywords:** colostrogenesis, energy, late gestation, performance, physiology

## Abstract

This study hypothesises that a high‐starch (HS) diet during the last month of gestation enhances colostrum yield and composition as well as the dam and goat kid metabolism, immune status and performance. Thirty multiparous and pregnant *Majorera* dairy goats were randomly assigned to a prepartum dietary treatment (CON vs. HS) on wk −4 relative to the expected parturition. Goats were fed either a control (*n* = 15; 25% DM of starch) or HS (*n* = 15; 32% DM of starch) diet during the last month of gestation. Blood samples were collected on wk −4, −3, −2 and −1 relative to expected parturition, immediately at parturition (d0) and on d 1, 2, 3, 5, 10, 15, 30 postpartum in dams and goat kids. Colostrum and milk yield were recorded, and gross chemical composition, somatic cell count (SCC), Brix degrees and immunoglobulin G (IgG) concentration were determined on d 0, 1, 2, 3, 5, 10, 15, 30 postpartum. In addition, dam and goat kid blood serum metabolites and plasma IgG concentrations were determined. Data was analysed using linear mixed models in SAS (SAS 9.4) including the prepartum diet, time (*T*), and the interaction between both as fixed effects. The statistical significance was set as *p* ≤ 0.05. The high‐starch diet did not have any effect either on colostrum nor on milk yield, gross chemical composition, SCC and Brix degrees. However, the excess of starch on the prepartum diet increased colostrum IgG concentration on d 0 (i.e. first milking) in the HS group compared to the control group (i.e. 85.4 and 61.2 mg/mL, respectively). Serum BHB concentration in the HS group increased progressively from wk −3 to parturition (i.e. 0.20 and 0.24 mmol/L, respectively), whereas the control group showed a sharp increase from wk −1 to parturition (i.e. 0.22 and 0.29 mmol/L, respectively). Calcium, total protein, lactate dehydrogenase, urea and albumin concentrations were lower in the HS group compared to the control group during the prepartum period. The high‐starch diet prepartum did not influence serum metabolites in the postpartum period except for lactate dehydrogenase, being lower in the first 5 days after parturition in the HS group. In the goat kids, body weight (BW), milk intake and plasma IgG concentrations were not affected by the high‐starch diet offered to the dams prepartum except for an increased serum total protein concentration observed on d 1 in the HS group compared to the CON group (i.e. 7.1 vs. 6.5 g/dL, respectively). The present results indicate that a high‐starch diet during the last month of gestation does not affect either colostrum yield or composition but increases colostrum IgG concentration, although it did not affect circulating IgG concentrations in goat kids from both groups. Based on the slower fat mobilisation in the HS group, high‐starch diets prepartum could promote a smoother transition from late gestation to early lactation in dairy goats. However, other metabolic variables should be considered in future studies.

**Abbreviations**
BHB: Beta‐hydroxybutyrate
BW: Body weight
DM: Dry matter
FFA: Free fatty acids
HS: High‐starch
LDH: Lactate dehydrogenase
LSM: Least Square Means
MI: Individual milk intake
NEB: Negative energy balance
RT: Rectal temperature
SCC: Somatic cell count
SEM: Standard error of the mean
*T*
: Time
TP: Total protein

## 1 Introduction

Studies on dairy and beef cattle have reported that prepartum nutrition can impact lactation and offspring performance (Keady et al. 2005; Chung et al. 2008; Moriel et al. 2021). Similar studies performed in sheep and goats have also suggested that prepartum nutritional management influences milk yield and shapes offspring health and performance (Sahlu et al. 1995; Shabrandi et al. 2019; Villar et al. 2023). During late gestation, dairy ruminants experience important metabolic adaptations due to the increased energy demands for foetal growth (Eley et al. 1978; Castagnino et al. 2015) while after parturition, they must increase energy intake to face the high energy requirements for milk synthesis. Cereal grains are one of the main components in ruminant diets, often used in combination with forage, to increase the energy supply. Starch is the main carbohydrate present in these cereal grains such as maize or wheat and is rapidly fermented by the rumen microbiota into propionate, which is absorbed into the bloodstream to be used as a glucose precursor (De Koster and Opsomer 2013).

To mitigate the negative energy balance (NEB) often seen in animals bearing twin or triplets, high‐starch (HS) diets are offered after parturition (Reynolds et al. 2001; Zamuner et al. 2020; Lunesu et al. 2021). However, it has been shown that feeding high‐starch diets during the last months of gestation can enhance gut adaptation to these highly fermentable diets and reduce fat mobilisation in dairy cows (Janovick et al. 2011), ewes (Banchero et al. 2004b) and goats (Celi et al. 2008). Besides these benefits, it has been demonstrated that energy intake modulates growth hormone and insulin‐like growth factor I secretion, which can enhance nutrient partitioning to the mammary gland and promote colostrum and milk synthesis (Banchero et al. 2015; Hare et al. 2023). Despite the scarce literature regarding the effects of starch supplementation on colostrum quality in small ruminants, few studies have reported promising results. For instance, Ramírez‐Vera et al. (2012) found higher colostrum yield in grazing crossbred goats fed 0.6 kg/day of flaked maize during the last 2 weeks prepartum. Similar findings were reported by Olivera‐Muzante et al. (2022), who found greater lactose concentrations and colostrum yields in Corriedale ewes supplemented with 0.5 kg/day of an energy‐protein lick. Additionally, some studies have focused on colostrum bioactive molecules suggesting that certain components such as immunoglobulins or oligosaccharides can be modulated by high‐starch diets provided during colostrogenesis (Mann et al. 2016; Fischer‐Tlustos et al. 2022). Further, this greater energy availability has been shown to have an impact on offspring viability by reducing neonatal mortality rates in grazing sheep fed whole maize during the last 2 weeks of gestation (Banchero et al. 2009).

Based on the above, there is still limited knowledge about the effects of high‐starch diets prepartum on colostrum yield and composition in dairy goats as well as the impact on their offspring. Therefore, this study hypothesises that feeding a high‐starch diet during the last month of gestation increases colostrum yield and enhances colostrum composition, and the metabolic and immune status of both dairy goats and goat kids. Therefore, the present study aimed to investigate the effects of a high‐starch diet prepartum on colostrum and milk yield and composition as well as on metabolism, immune status and performance of dams and goat kids.

## 2 Materials and Methods

All experimental procedures were approved by the Ethical Committee for Animal Experimentation of the Universidad de Las Palmas de Gran Canaria (OEBA‐ULPGC 03/2023; OEBA‐ULPGC 04/2023).

### 2.1 Experimental Design

Thirty multiparous *Majorera* dairy goats (average milk yield 466 ± 198 kg/lactation) in their second to fourth lactation, housed in a free‐stall barn, were used in this study. The *Majorera* breed, an autochthonous goat from the Canary Islands, was selected as a representative high‐yield dairy goat due to its strong genetic selection for milk production. Groups were balanced by body weight (*BW*; i.e. 64.6 ± 1.14 kg) and the animals were fed either a control (*n* = 15 [*CON*]; 25% starch based on dry matter [DM]) or a high‐starch (*n* = 15 [*HS*]; 32% starch based on DM) diet during the last month of gestation. Control diet was formulated following the guidelines provided by the *L'Institut National de la Recherche Agronomique* (INRA; Sauvant et al. 2018) to meet the 100% energy requirements for dry goats in the last month of gestation (Table 1). In a previous pilot study, the average daily intake and maize refusals were recorded in order to determine the excess of dietary starch accepted by the gravid animals. In brief, it was observed that *Majorera* pregnant dairy goats that were offered a diet containing 50% starch (DM basis) daily could only eat 32% starch (DM basis) on average. Therefore, the final experimental diets were formulated to provide 25% and 32% starch (DM basis) in the CON and HS diets, respectively (Table 2). Additionally, both control and high‐starch diets were intended to be isonitrogenous, although there were minor variations in crude protein between the control (61.1 g) and the high‐starch (65.4 g) diets. Each animal was individually fed concentrate divided in two meals (50% each meal, at 07:30 AM and 17:00 PM) while forage was provided daily in the free stall. Additionally, all the animals had access to fresh water and mineral blocks (i.e. Na, 36.0 g/kg; Ca, 1.00 g/kg; Mg, 0.6 g/kg; MnSO_4_, 312 mg/kg FeSO_4_, 200 mg/kg; Na_2_SeO_3_, 33 mg/kg; Ca (IO_3_), 24 mg/kg; (CH_3_COO)_2_Co*_4_H_2_O, 8 mg/kg). After parturition, both groups received a diet to meet the requirements for dairy goats in the first month of lactation (Table 1). Dams were milked once daily at 07:30 h in a double 12‐stall parallel milking parlour (Alfa Laval Iberia SA, Madrid, Spain) equipped with recording jars (3.5 L ± 5%) using the procedure described by Torres et al. (2013). Single and twin‐born goat kids with a birth BW > 2.5 kg (*n* = 43) were immediately separated from their dams after birth and allocated to either the HS group (*n* = 25) or the CON group (*n* = 18) depending on the experimental group of the dam. Goat kids were bottle‐fed with their dam first‐milking colostrum (i.e. 10% of the birth BW) divided in two meals within the first 12 h of life (i.e. at 2 and 12 h relative to birth) according to Argüello et al. (2004). After that, animals were fed twice daily with bulk tank goat milk using teat feeding buckets. The health status of both dairy goats and goat kids was monitored once daily.

**Table 1:** Daily nutritional requirements for goats in 4th and 5th month of gestation and 1st of lactation according to INRA (Sauvant 2018).

	Gestation		Lactation
Requirements	4th month	5th month	1st month
UEL	0.97	0.99	1.83
UFL	1.25	1.51	1.80
PDI, g	62.0	99.5	165
Calcium, g	3.24	4.75	6.75
Phosphorous, g	3.84	5.35	13.75

Abbreviations: PDI, Protéines Digestibles dans l'Intestin (Digestible protein in the intestine); UEL, Unité d'Encombrement Lait (Fill Unit for Dairy); UFL, Unité Fourragère Lait (Forage Unit for Lactation).

**Table 2:** Ingredients and chemical composition of experimental diets.

	Gestation			
		5th month		Lactation
Group	4th^th^ month	CON	HS	1st^st^ month
Ingredients, kg (dry matter basis)				
Maize	0.52	0.48	0.65	0.56
Soybean meal (oil < 5%, 46% protein + oil)	—	0.09	—	0.10
Lucerne dehydrated (16%–18% protein)	—	—	—	0.16
Italian ryegrass hay	0.51	0.85	0.85	0.51
Lucerne hay	—	—	—	0.43
Nutrients (dry matter basis)				
Gross energy, kcal	4 813.0	6 143.1	6 489.1	7 457.8
UEL	0.93	1.05	1.10	1.75
UFL	1.25	1.52	1.65	1.78
PDI, g	85	115	118.5	167
Crude protein, g	53.1	61.1	65.4	96.5
Crude fat, g	36.6	41.5	46.9	51.1
NDF, g	402.8	620.1	628.3	680.1
Starch, g	439.7	356.2	478.1	425.6
Calcium, g	2.1	3.6	3.3	7.8
Phosphorous, g	2.7	3.6	3.5	4.3

Abbreviations: CON, control group; HS, high‐starch group; NDF, neutral detergent fibre; PDI, Protéines Digestibles dans l'Intestin (Digestible protein in the intestine); UEL, Unité d'Encombrement Lait (Fill Unit for Dairy); UFL, Unité Fourragère Lait (Forage Unit for Lactation).

### 2.2 Data and Sample Collection

The experiment started at wk −4 relative to expected parturition and finished on wk 4 postpartum. Throughout the study, the amount of concentrate offered and refused were measured individually and daily after the second meal using a digital scale (Comfort 300 Slim, Soehnle, Germany) whereas dams BW was recorded weekly using a digital scale (EOS150K50XL, KERN & SOHN GmbH, Germany). Blood samples from the dams were collected from the jugular vein using 10 mL syringes (Injekt Braun, Braun, Germany) and 20 G needles (Sterican Braun, Braun, Germany) on wk −4, −3, −2, −1 relative to expected parturition, immediately at parturition (d 0) and then on d 1, 2, 3, 5, 10, 15 and 30 postpartum. In the goat kids, blood samples were always collected before feeding and following the same sampling scheme as the dams postpartum. Blood was immediately transferred to EDTA‐K2 tubes (BD Vacutainer, UK) for plasma collection, and serum tubes (SEROTUB, DeltalabTM, Spain) to obtain blood serum. Plasma tubes were centrifuged at 2190 × *g* for 10 min at 4°C (Hettich Zentrifugen, Universal 32R, Tuttlingen, Germany) while serum tubes were stored at room temperature for 2 h and then centrifuged at 2190 × *g* for 10 min at 25°C. Both plasma and serum were aliquoted in 1.5 mL Eppendorf tubes and stored at −20°C until further analysis. First milking colostrum was collected within the first 2 h after parturition (i.e. d 0) followed by transition and mature milk (i.e. d 1, 2, 3, 5, 10, 15 and 30 relative to parturition) collection. The present classification was based on previous data reported by Sánchez‐Macías et al. (2014), although it must be noted that the compositional differences between colostrum and transition milk are highly influenced by the milking frequency. Animals were milked and colostrum and milk yields were recorded. Individual milk intake (*MI*), rectal temperature (*RT*) and BW of goat kids were recorded at birth and on d 5, 10, 15 and 30 of life.

### 2.3 Sample Analysis

Blood, colostrum and milk samples were analysed for IgG concentration using a commercial ELISA kit (Bethyl Laboratories, Montgomery, TX, USA). The intra‐assay and inter‐assay coefficients of variation were 4.41% and 4.86%, respectively. Concentrations of beta‐hydroxybutyrate (*BHB*; 137019910930, DiaSys Diagnostics, Holzheim, Germany), calcium (GN12125, RAL laboratories, Barcelona, Spain), free fatty acids (*FFA*; 157819910935, DiaSys Diagnostics, Holzheim, Germany), glucose (GN45126, RAL laboratories, Barcelona, Spain), lactate dehydrogenase (*LDH*; GN42125, RAL laboratories, Barcelona, Spain), total proteins (*TP*; GN46125, RAL laboratories, Barcelona, Spain), albumin (GN86125, RAL laboratories, Barcelona, Spain) and urea (GN70125, RAL laboratories, Barcelona, Spain) were measured in blood serum using an automatic spectrophotometer (METROLAB 2300GL, RAL laboratories, Barcelona, Spain). The intra‐assay coefficients of variation were 0.56%, 2.10%, 1.10%, 1.92%, 1.07%, 0.90%, 0.50% and 2.79%, respectively. The inter‐assay coefficients of variation were 2.15%, 3.09%, 2.16%, 3.10%, 1.15%, 1.43%, 0.80% and 2.65%, respectively.

Colostrum and milk gross chemical composition (i.e. fat, protein, lactose and total solids) were determined by infra‐red spectroscopy using a MilkoScan Mars (FOSS IBERIA, Spain). Colostrum samples were diluted 1:4 (v/v) using deionized water based on the procedure described by Spina et al. (2021). The somatic cell count (*SCC*) in colostrum and milk was determined using a DeLaval cell counter (DeLaval, Tumba, Sweden). Samples with SCC greater than 3000 × 10^3^ cells/mL were diluted 1:5 (v/v) with saline solution as described by Kawai et al. (2013). In addition, a digital refractometer (PAL‐1, ATAGO CO., LTD., Tokio, Japan) was used to determined Brix° in colostrum and milk samples.

### 2.4 Statistical Analysis

The POWER procedure of SAS (version 9.4, SAS Institute Inc., Cary, NC, USA) determined 13 animals per group as the minimum number of animals required to set a significant effect on colostrum IgG concentration (differences between groups > 6.8 mg of IgG per mL) based on previous literature (41.1 mg/mL with a SD of 5.65 mg/mL; Hernández‐Castellano et al. 2016). The significance level was set as 5% and the power as 80%.

The residual distributions of the data were assessed using the UNIVARIATE procedure of SAS (SAS 9.4). Data that did not follow normal distributions were log‐transformed (log10) to get a normal distribution of residues and homogeneity. Results are presented as Least Square Means (*LSM*) ± standard error of the mean (*SEM*) and the Bonferroni test was used to determine significant differences (*p* ≤ 0.05). Prepartum and postpartum data regarding blood parameters were analysed separately using the MIXED procedure of SAS (SAS 9.4). Data on wk −4 was set as the baseline for the prepartum data (wk −3, −2, −1 and d 0) and d 0 was set as the baseline for the postpartum data (d 1, 2, 3, 5, 10, 15 and 30). Therefore, the model included diet (HS vs. CON), time (wk −3 to d 0 for prepartum data and d 1 to d 30 for postpartum data) and the interaction between both (Diet × Time) as fixed effects. The animal (i.e. goat or goat kid) was considered the individual subject, time as a repeated measure and litter size as a random effect.

## 3 Results

Throughout the study, three dams were removed due to health problems, including one abortion (i.e. 2 and 1 from the HS and CON, respectively). Therefore, the final experimental size for each group was HS *n* = 13 and CON *n* = 14. No differences in the concentrate DM intake were observed between groups on the 4th month of gestation and 1st month of lactation. In addition, concentrate DM intake in the fifth month of gestation differed between groups, with the HS exhibiting greater DM intake compared to the CON group (622.5 ± 12.60 g/day and 516.5 ± 11.17 g of DM/day, respectively; *p* < 0.001). Gestation length and litter size showed no differences between groups (150 ± 1.3 days and 1.68 ± 0.10 kids, respectively). Similarly, no differences in birth BW (i.e. 3.1 ± 0.14 kg) were observed between groups.

### 3.1 Blood Metabolites in Dairy Goats Prepartum

**Figure 1:**
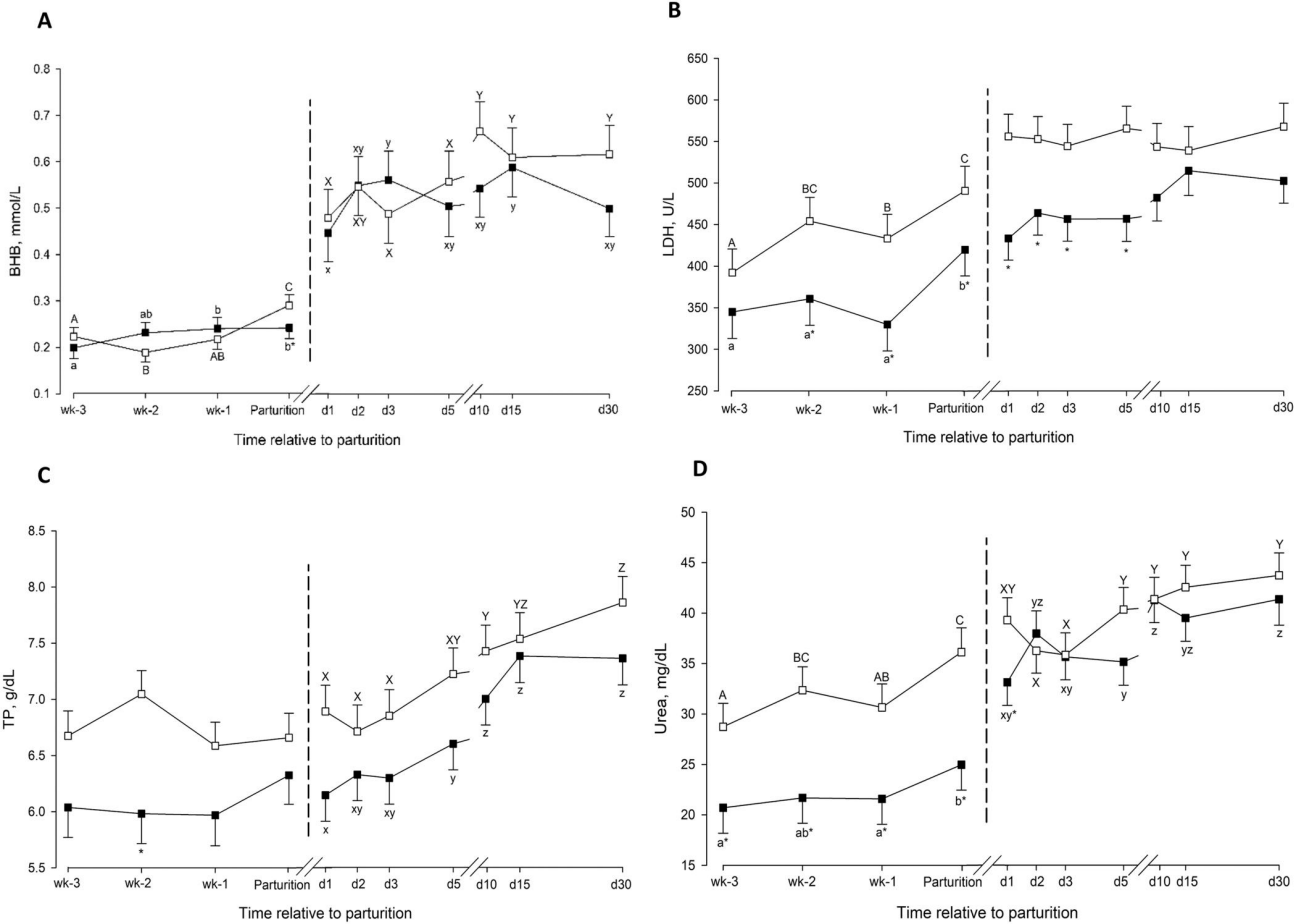
Serum beta‐Hydroxybutyrate (BHB; A), lactate dehydrogenase (LDH; B), total protein (TP; C) and urea (D) concentration in the HS (■; 32% starch [DM basis], *n* = 13) and CON (□; 25% starch [DM basis], *n* = 14) groups during the prepartum (wk −3, −2, −1 and parturition), postpartum period (d 1, 2, 3, 5, 10, 15 and 30 relative to parturition). Different superscripts (a–c) indicate significant differences (*p* < 0.05) in the HS group during the prepartum period. Different superscripts (A–C) indicate significant differences (*p* < 0.05) in the CON group during the prepartum period. Different superscripts (x‐z) indicate significant differences (*p* < 0.05) in the HS group during the postpartum period. Different superscripts (X‐Z) indicate significant differences (*p* < 0.05) in the CON group during the postpartum period. Significant differences between both groups are represented with (*). Data is expressed as LSM ± SEM.

The effects of the HS diet on prepartum blood metabolites concentrations are shown in Table 3. Plasma IgG concentration was not affected either by the prepartum diet (*p* = 0.533) or time (*p* = 0.723). A two‐way interaction was observed for serum BHB (Figure 1A; *p* = 0.006). The BHB concentration in the HS group increased constantly from wk −3 to parturition (0.20 and 0.24 mmol/L, respectively) whereas BHB concentration decreased from wk −3 to wk −2 (0.22 and 0.19 mmol/L, respectively) and then increased until parturition (0.29 mmol/L) in the CON group. Calcium and TP concentrations were affected by the prepartum diet (*p* ≤ 0.047). The HS group showed lower calcium and TP concentrations (7.1 mg/dL and 6.1 g/dL, respectively) than the CON group (7.6 mg/dL and 6.7 g/dL, respectively) during the entire prepartum period (wk −3 to parturition). In contrast, serum FFA concentration was not affected by the prepartum diet (*p* = 0.793) but was affected by time (*p* < 0.001) increasing from wk −3 to parturition (0.25 and 0.52 mmol/L, respectively). Glucose concentration was not affected by either the prepartum diet (*p* = 0.135) or time (*p* = 0.938). However, LDH, albumin and urea concentrations were affected by both the prepartum diet (*p* ≤ 0.039) and time (*p* ≤ 0.007). Serum LDH, albumin and urea concentrations were lower in the HS group (362.7 U/L, 3.0 g/dL and 22.2 mg/dL, respectively) compared to the CON group (441.5 U/L, 3.3 g/dL and 32.0 mg/dL, respectively). In addition, LDH, albumin and urea concentrations in both groups increased progressively from wk −3 (367.4 U/L, 3.1 g/dL and 24.7 mg/dL, respectively) to parturition (454.1 U/L, 3.2 g/dL and 30.6 mg/dL, respectively).

**Table 3:** Goat serum metabolites and plasma IgG concentration in the HS (32% starch [DM basis], *n* = 13) and CON (25% starch [DM basis], *n* = 14) groups during the prepartum period (wk −3, −2, −1) and at parturition (d 0).

	Groups			Fixed effects		
Variables	HS	CON	SEM	Diet	Time	Diet × *T*
BHB, mmol/L	0.23	0.23	0.02	0.832	< 0.001	0.006
Glucose, mg/dL	43.8	40.4	2.52	0.135	0.938	0.467
FFA, mmol/L	0.34	0.35	0.04	0.793	< 0.001	0.583
Calcium, mg/dL	7.1	7.6	0.16	0.047	0.299	0.606
LDH, U/L	362.7	441.5	23.81	0.039	< 0.001	0.256
TP, g/dL	6.1	6.7	0.16	0.019	0.110	0.147
Urea, mg/dL	22.2	32.0	2.25	< 0.001	< 0.001	0.458
Albumin, g/dL	3.0	3.3	0.07	0.017	0.007	0.347
IgG, mg/mL	17.1	18.0	2.70	0.533	0.723	0.406

Abbreviations: BHB, beta‐hydroxybutyrate; CON, control group; FFA, free fatty acids; HS, high‐starch group; IgG, immunoglobulin G; LDH, lactate dehydrogenase; *T*, time; TP, total protein.

### 3.2 Blood Metabolites in Dairy Goats Postpartum

The effects of the HS diet on postpartum blood metabolites concentrations are shown in Table 4. Plasma IgG concentration was not affected by the prepartum diet (*p* = 0.414) but was affected by time (*p* < 0.001), increasing gradually from d 1 to d 30 (18.2 and 21.1 mg/mL, respectively). Similarly, BHB, calcium, TP and albumin concentrations were not affected by the prepartum diet (*p* ≥ 0.093) but were affected by time (*p* ≤ 0.019). Serum BHB concentrations increased from d 1 to d 2 (0.5 and 0.6 mmol/L, respectively), decreasing on d 3 (0.5 mmol/L) and then increased progressively until d 30 (0.6 mmol/L). Calcium concentration increased constantly from d 1 to d 30 (7.2 and 7.9 mg/dL, respectively), as well as TP (6.5 and 7.6 g/dL, respectively) and albumin concentrations (3.1 and 3.4 g/dL, respectively). In contrast, LDH concentration was not affected by time (*p* = 0.456) but was affected by the prepartum diet (*p* = 0.019) being lower in the HS group than in the CON group. An interaction between the prepartum diet and time (*p* = 0.028) was observed for urea concentration. In the HS group, urea concentration increased from d 1 to d 2 (33.1 and 37.9 mg/dL, respectively), decreasing until d 5 (35.1 mg/dL) and increasing again until d 30 (41.3 mg/dL), whereas the CON group decreased from d 1 to d 3 (39.3 and 35.8 mg/dL, respectively) and then increased progressively until d 30 (43.7 mg/dL). Glucose and FFA concentrations were not affected by either the prepartum diet (*p* ≥ 0.304) or time (*p* ≥ 0.154).

**Table 4:** Goat serum metabolites and plasma IgG concentrations in the HS (32% starch [DM basis], *n* = 13) and CON (25% starch [DM basis], *n* = 14) groups during the postpartum period (d 1, 2, 3, 5, 10, 15 and 30).

	Groups		SEM	Fixed effects		
Variables	HS	CON		Diet	Time	Diet × *T*
BHB, mmol/L	0.5	0.6	0.05	0.578	0.019	0.281
Glucose, mg/dL	42.2	44.6	2.89	0.304	0.163	0.217
FFA, mmol/L	0.5	0.5	0.08	0.750	0.154	0.200
Calcium, mg/dL	7.3	7.7	0.32	0.176	< 0.001	0.220
LDH, U/L	473.0	552.8	22.23	0.019	0.456	0.323
TP, g/dL	6.7	7.2	0.20	0.093	< 0.001	0.408
Urea, mg/dL	37.7	39.9	1.87	0.270	< 0.001	0.028
Albumin, g/dL	3.1	3.3	0.13	0.105	< 0.001	0.126
IgG, mg/mL	18.8	20.0	1.00	0.414	< 0.001	0.375

Abbreviations: BHB, beta‐hydroxybutyrate; CON, control group; FFA, free fatty acids; HS, high‐starch group; IgG, immunoglobulin G; LDH, lactate dehydrogenase; *T,* time; TP, total protein.

### 3.3 Colostrum and Milk Outcomes

The effects of the HS diet on colostrum and milk outcomes are summarised in Table 5. Colostrum and milk yield, gross chemical composition, SCC and Brix degrees were not affected by the high‐starch diet (*p* ≥ 0.461). However, an interaction between diet and time was observed for the IgG concentration (Figure 1; *p* = 0.024). A higher IgG concentration on d 0 was observed in the HS group compared to the CON group (85.4 and 61.2 mg/mL, respectively). Besides this, the other parameters evaluated in colostrum and milk were affected by time (*p* ≤ 0.006). Yield increased from d 0 to d 30 (1.8 and 2.5 kg/day, respectively) as well as lactose percentage (2.8% and 4.6%, respectively). In contrast, fat, protein and total solids percentages decreased from d 0 (7.9, 14.4% and 27.7%, respectively) to d 30 (4.2, 3.79% and 13.4%, respectively). Similarly, Brix degrees decreased from d 0 (24.4°) to d 30 (9.3°) whereas SCC increased from d 0 to d 1 (2.8 and 3.0 log_10_ cells/mL, respectively) and decreased progressively until d 30 (2.4 log_10_ cells/mL).

**Table 5:** Yield, gross chemical composition, SCC, Brix degrees and IgG concentration in colostrum and milk of goats from the HS (32% starch [DM basis], *n* = 13) and CON (25% starch [DM basis], *n* = 14) groups during the first month postpartum.

		Days relative to parturition									Fixed effects		
	Group	0	1	2	3	5	10	15	30	SEM	Diet	Time	Diet × *T*
Yield, kg/d	HS	1.9^ab^	1.4^a^	1.8^ab^	2.1^ab^	2.4^b^	2.5^b^	2.4^b^	2.6^b^	0.10	0.759	< 0.0001	0.981
	CON	1.7^ab^	1.4^a^	1.9^ab^	2.0^ab^	2.2^ab^	2.3^ab^	2.6^b^	2.5^b^				
Lactose, %	HS	2.9^a^	3.7^b^	4.0^c^	4.2^c^	4.5^d^	4.6^d^	4.7^d^	4.6^d^	0.06	0.827	< 0.0001	0.617
	CON	2.8^a^	3.5^b^	4.2^c^	4.3^c^	4.6^d^	4.6^d^	4.8^d^	4.7^d^				
Fat, %	HS	8.7^a^	6.2^b^	6.4^b^	6.0^b^	5.6^bc^	4.4^bc^	3.7^c^	4.0^bc^	0.20	0.516	0.006	0.001
	CON	7.0^a^	6.9^a^	6.7^a^	6.7^a^	5.4^ab^	4.5^b^	4.0^b^	4.5^b^				
Protein, %	HS	14.5^a^	8.3^b^	6.7^bc^	5.9^bc^	5.0^c^	4.1^c^	3.9^c^	3.7^c^	0.27	0.489	< 0.0001	0.879
	CON	14.4^a^	9.6^b^	7.4^c^	5.9^cd^	5.0^cd^	4.0^d^	4.1^d^	3.7^d^				
Total solids, %	HS	28.5^a^	20.1^b^	18.3^bc^	17.0^bc^	15.7^bc^	14.0^c^	13.3^c^	13.1^c^	0.38	0.461	< 0.0001	0.245
	CON	26.8^a^	22.4^b^	19.2^c^	17.5^cd^	15.7^cd^	13.9^d^	13.8^d^	13.7^d^				
SCC, log_10_ cells/mL	HS	2.7^a^	3.0^a^	3.0^a^	2.7^a^	2.7^a^	2.5^a^	2.3^a^	2.3^a^	0.09	0.774	0.001	0.845
	CON	2.9^a^	3.0^a^	3.0^a^	2.8^a^	2.6^a^	2.4^a^	2.5^a^	2.7^a^				
Brix, °	HS	25.1^a^	17.5^b^	13.2^c^	11.8^c^	11.5^c^	9.6^c^	9.5^c^	9.3^c^	0.42	0.526	< 0.0001	0.482
	CON	23.8^a^	18.4^b^	15.6^bc^	12.8^cd^	11.3^d^	9.8^d^	9.5^d^	9.2^d^				
IgG, mg/mL	HS	85.4^a*^	37.0^b^	13.9^c^	8.6^c^	2.5^c^	1.7^c^	1.4^c^	1.1^c^	1.77	0.100	< 0.0001	0.024
	CON	61.2^a^	30.7^b^	11.5^c^	5.5^c^	3.8^c^	2.3^c^	1.7^c^	1.7^c^				

*Note:* Data is expressed as LSM ± SEM. Different superscripts (a–d) indicate significant mean differences within a row. Significant differences between groups are represented with (*).

Abbreviations: CON, control group; HS, high‐starch group; IgG, immunoglobulin G; SCC, somatic cell count; *T*, time.

### 3.4 Blood Metabolites and Performance in Goat Kids

The effects of the HS diet on goat kid metabolism and performance are presented in Table 6. Body weight and MI were not affected by the prepartum diet of the dam (*p* ≥ 0.338). However, both MI and BW increased from d 5 (862.0 mL and 3.4 kg, respectively) to d 30 (1418 mL and 7.0 kg, respectively; *p* < 0.001). Similarly, rectal temperature was not affected by the prepartum diet of the dam (*p* = 0.150) but was affected by time (*p* < 0.001), increasing from birth to d 10 (38.5°C and 39.4°C, respectively) and decreasing slightly on d 30 (39.2°C).

**Table 6:** Body weight, individual milk intake, rectal temperature, serum metabolites and plasma IgG concentrations in goat kids born from goats from the HS (32% starch [DM basis], *n* = 25) and CON (25% starch [DM basis], *n* = 18) groups.

	Groups		SEM	Fixed effects		
Variables	HS	CON		Diet	Time	Diet × *T*
BW, kg	4.5	4.4	0.28	0.741	< 0.001	0.440
MI, mL	975.8	907.1	59.76	0.338	< 0.001	0.087
RT, °C	39.1	39.2	0.05	0.150	< 0.001	0.929
Glucose, mg/dL	71.8	73.2	2.74	0.674	< 0.001	0.862
Calcium, mg/dL	10.4	10.3	0.13	0.468	< 0.001	0.090
LDH, U/L	773.5	832.7	31.38	0.117	< 0.001	0.780
TP, g/dL	6.1	5.9	0.11	0.232	< 0.001	0.011
Urea, mg/dL	44.3	45.5	1.13	0.507	< 0.001	0.288
Albumin, g/dL	2.8	2.8	0.05	0.490	< 0.001	0.070
IgG, mg/mL	12.7	12.4	0.93	0.796	< 0.001	0.166

*Note:* HS = goat kids from dams fed a high‐starch diet prepartum (134% DM starch requirements); CON = goat kids from dams fed a control diet prepartum (100% DM starch requirements).

Abbreviations: IgG, immunoglobulin G; LDH, lactate dehydrogenase; MI, individual milk intake; RT, rectal temperature; *T*, time; TP, total protein.

**Figure 2:**
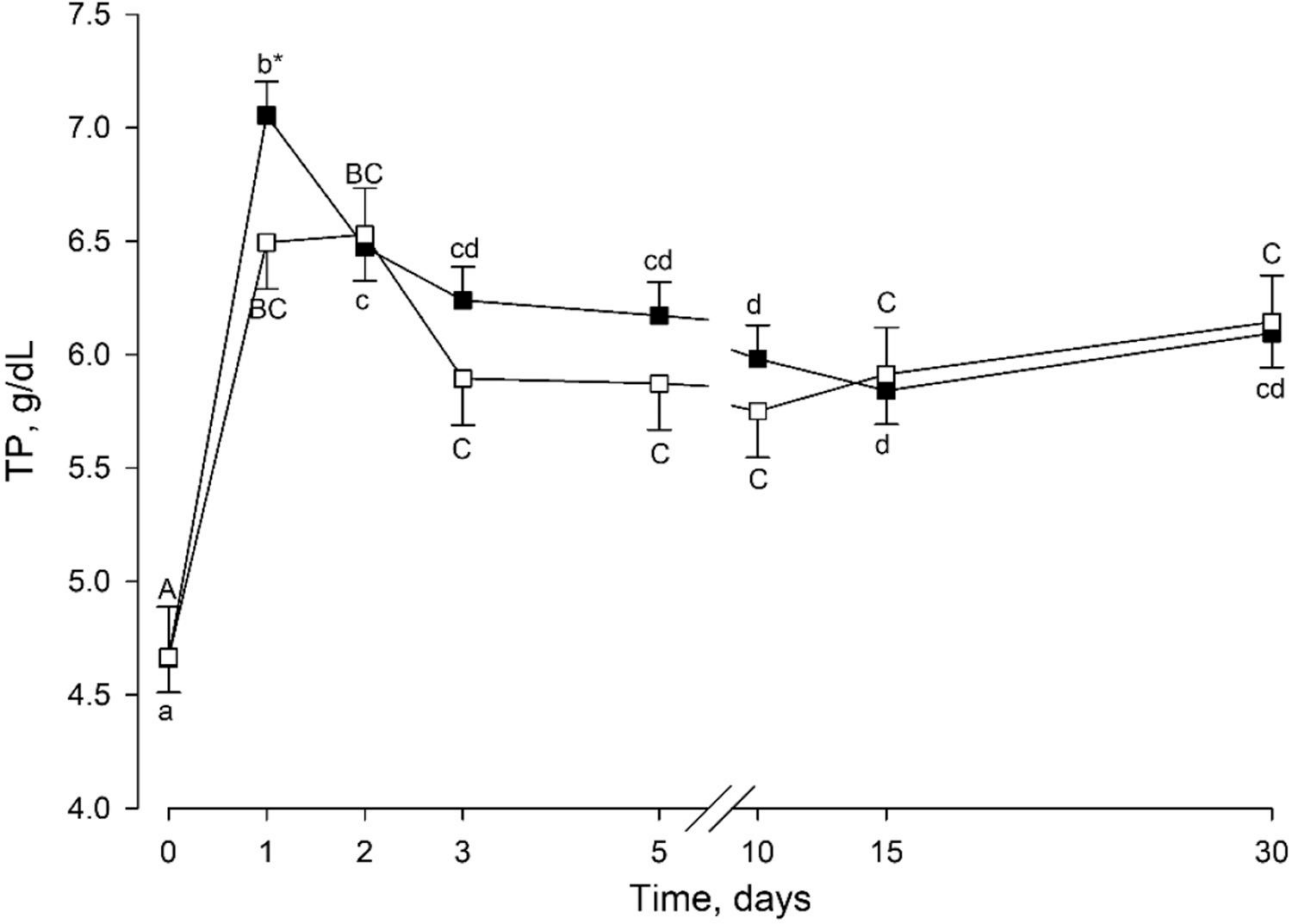
Serum total protein concentration in goat kids born from HS goats (■; *n* = 25) and CON goats (□; *n* = 18) throughout the experimental period. Different superscripts (a–d) indicate significant differences (*p* < 0.05) in the HS group. Different superscripts (A–C) indicate significant differences (*p* < 0.05) in the CON group. Significant differences between both groups are represented with (*). Data is expressed as LSM ± SEM. TP, total protein.

A two‐way interaction between diet and time was observed for serum TP concentration (Figure 2; *p* = 0.011). Total protein increased from d 0 to d 1 (4.7 and 7.1 g/dL, respectively) decreasing progressively until d 15 (5.8 g/dL) and increasing again until d 30 (6.1 g/dL) in goat kids from the HS group. The CON group showed increased TP concentrations from d 0 to d 2 (4.7 and 6.5 g/dL, respectively), decreasing progressively until d 10 (5.8 g/dL) and increasing again until d 30 (6.1 g/dL). The rest of the serum variables (i.e. calcium, glucose, LDH, albumin and urea) were not affected by the prepartum diet of the dam (*p* ≥ 0.117) but were affected by time (*p* < 0.001). Albumin concentration decreased from d 0 to d 3 (2.9 and 2.5 g/dL, respectively) and then increased progressively until d 30 (3.5 g/dL). Similarly, calcium concentration decreased from d 0 to d 2 (11.1 and 10.1 mg/dL, respectively), then increased to d 5 (10.5 mg/dL) and decreased again until d 30 (9.8 mg/dL). Lactate dehydrogenase concentration increased from d 0 to d 1 (776.0 and 849.2 U/L, respectively), decreased on d 3 (558.3 U/L) and then increased progressively until d 30 (969.4 U/L). Similarly, urea concentration increased from d 0 to d 1 (43.1 and 62.7 mg/dL, respectively), decreasing until d 30 (40.1 mg/dL). In contrast, glucose concentration increased from d 0 to d 30 (12.4 and 105.3 mg/dL, respectively). Similarly, plasma IgG concentration was not affected by the prepartum diet of the dam (*p* = 0.796) but was affected by time (*p* < 0.001), increasing from d 0 to d 2 (0.7 and 15.7 mg/mL) to decrease progressively until d 30 (6.2 mg/mL).

## 4 Discussion

It is well established that dietary modifications during the prepartum period can affect milk yield, metabolism and offspring performance of dairy and beef cattle (Haisan et al. 2021; Hare et al. 2023), ewes (Banchero et al. 2004b) and goats (Celi et al. 2008). However, this is the first study exploring the effects of a high‐starch diet prepartum on dairy goats and their offspring under an intensive management system.

According to previous studies, exceeding energy requirements during the last stage of gestation can increase glucose and insulin concentrations (Banchero et al. 2004a; Haisan et al. 2021; Janovick et al. 2011) which, in turn, may increase energy availability and promote a more efficient colostrum synthesis. In the present study, a high‐starch diet prepartum did not cause an increased serum glucose concentration during the prepartum period. In addition, it did not affect either colostrum yield or lactose percentage. Therefore, as no differences were observed in circulating glucose concentrations between groups, the lack of effects on colostrum yield and lactose percentage were also expected. In agreement with these findings, Ramírez‐Vera et al. (2012) reported no differences on plasma glucose concentration in grazing pregnant goats supplemented with maize compared to those that were not supplemented under the same grazing conditions. This lack of response to energy supplementation is likely associated with inherent metabolic adaptations over the gestation period aiming to regulate glucose metabolism. Indeed, Hare et al. (2023) suggested that prepartum glucose partitioning to the mammary gland in dairy cattle may already be increased during colostrogenesis, so the metabolic pathways involving glucose could be saturated before the energy supplementation. In support of this idea, some studies have reported that high yielding breeds are genetically programmed for body tissue mobilisation in the transition period regardless of the nutritive value of the diet (Drackley et al. 2014; Ribeiro et al. 2023). Given that certain dairy goat breeds have been selected for milk production, these metabolic adaptations, mainly described in cattle, could also explain the lack of effect on circulating glucose concentrations observed in this study.

Regarding other metabolites, increased serum BHB concentration prepartum has been previously reported by Zamuner et al. (2020) in response to the high energy demands during late gestation in Saanen goats. Despite the present study showed increased BHB concentration in both groups before parturition, goats fed the high‐starch diet prepartum increased BHB concentrations in a lower extent than the goats receiving the control diet. These results suggest that providing a high‐starch diet prepartum can promote a smoother transition to lactation, reducing the extent of energy reserves mobilisation before parturition, contributing not only to reducing the effects of NEB, but also promoting better gut adaptation to high fermentable lactation diets. However, this cannot be fully demonstrated as FFA concentrations did not differ between groups which indicates that despite the effects on BHB concentrations, these animals were not under intense catabolism. Therefore, it is likely that a diet supplementation based on 32% DM starch prepartum may not be sufficient to enhance dam metabolic status during the last weeks of gestation. Future studies should include other metabolic variables (e.g. insulin and leptin concentrations) and gene expression to deeply characterise the effects of high‐starch diets on the animal metabolism.

Despite both experimental diets were almost isonitrogenous, the HS group showed reduced TP, urea and albumin concentrations. This could be attributed to different protein sources in the prepartum diets, as the CON group received 100 g/day of soybean meal as part of the concentrate while the HS group was fed maize exclusively. These results agree with previous studies showing increased plasma urea concentrations in Saanen goats fed soybean meal compared to goats fed other protein sources (Santos et al. 2014). In addition, increased dietary starch content may impact ruminal and hind‐gut microbiota (Khafipour et al. 2016; Rivera‐Chacon et al. 2024) affecting fermentation and nutrient digestibility and, consequently, altering the absorption of aminoacids, FFA and other substrates. Further, according to Emmanuel and Edjtehadi (1981), high levels of ammonia in the bloodstream may damage cell membranes leading to impaired transport of nutrients and consequently interfere with cell metabolism. This might explain not only TP and urea differences between groups but also the greater LDH concentration observed in the CON group during the pre‐ and postpartum periods.

Whether exceeding dietary energy requirements prepartum affects or not colostrum IgG concentrations is still controversial. While Mann et al. (2016) and Hare et al. (2023) reported lower colostrum IgG concentrations in dairy and beef cattle fed high energy diets during the last month of gestation, Gallo et al. (2020) found that exceeding dietary energy requirements (i.e. 10% ME kg/DM) did not affect colostrum immunoglobulin concentration in crossbreed ewes. Despite this, there is still limited literature on the effects of high‐starch diets on colostrum IgG concentration in dairy goats. Changes on colostrum IgG concentrations can be likely associated with either modifications in plasma IgG concentrations and impaired IgG transfer from blood to colostrum (Mann et al. 2016) or modifications on the IgG local synthesis (Sordillo and Nickerson 1988; Aitken et al. 2011). However, it is still unknown which specific mechanisms regulate these physiological pathways as colostrum IgG concentration has been demonstrated to be highly variable between individual animals (Baumrucker and Bruckmaier 2014). Although the present study did not focus on the molecular mechanisms regulating colostrum bioactive components, high energy diets are likely to modify glucose metabolism, potentially inducing hormonal changes that can impact key pathways involved in colostrogenesis such as the synthesis and transcytosis of immune components. For instance, increased glucose concentrations have been shown to alter B cells function, decreasing immunoglobulin secretion under in‐vitro experimental conditions (Jennbacken et al. 2013). However, since B cells can express insulin receptors (Baumrucker et al. 2022), it can be also hypothesised that high energy diets may impact their function systemically and within the mammary gland during colostrogenesis.

Despite the benefits of providing high‐starch diets at the onset of lactation, abrupt changes in the dietary starch content during the transition period increases the risk of acute rumen acidosis, reducing ruminal fibre digestibility, ruminal acetate/propionate ratio, and increases the concentration of circulating inflammation markers which in turn can cause reduced dry matter intake and milk yield in dairy cows (Emmanuel et al. 2008; Haisan et al. 2021; Hernández‐Castellano et al. 2021), ewes and goats (Shen et al. 2018; Zhang et al. 2024). Since the present study found no effects on colostrum yield or chemical composition, no differences in milk yield and composition were expected either. In fact, there are discrepancies in the literature regarding the effects of starch supplementation on milk yield and composition. For example, Tsiplakou et al. (2017) found that feeding high‐starch diets to dairy goats at d 90 of lactation has no effects on milk chemical composition nor in casein profile, whereas Bernard et al. (2022) reported a reduction in milk fat content of goats fed a high concentrate diet. In contrast, a reduction of dietary fibre content followed by the inclusion of cereal grains has been shown to increase goats milk yield (Pulina et al. 2007). Therefore, it can be assumed that the inconsistency among studies is likely attributed to multiple factors such as different starch sources and inclusion level, timing and length of supplementation, as well as breed and management system, factors that should be deeply assessed in future studies.

Regarding goat kid metabolism and performance, some studies have reported no effects of energy supplementation in the last weeks of gestation on lamb and goat kid birth BW (Banchero et al. 2004b; Hall et al. 1992). However, some authors have suggested that glucose uptake by the foetus is directly proportional to the available glucose present in the dam bloodstream, leading to heavier newborns (Battaglia and Meschia 1988; Banchero et al. 2004a). Agreeing with this, Elkhair et al. (2020) found greater birth BW (i.e. 3.2 vs. 2.0 kg) in goat kids born to Egyptian Nubian goats supplemented daily with 200 g of maize starch and 100 g of molasses. Since dietary starch intake did not alter glucose availability in the dams, differences in the offspring birth BW were not expected in the present study. Additionally, the prepartum diet of the dam did not have any impact on milk intake, agreeing with a previous study performed in Red Syrian goats (Celi et al. 2008). Therefore, it seems that the high‐starch diet prepartum provided to the dams does not influence offspring growth, which can be possibly associated with a controlled supply of metabolic substrates through the placenta (Cowell et al. 2023) or a limited efficiency of feed utilisation.

In connection with the above, goat kids born from HS goats showed higher TP concentration after colostrum intake whereas other metabolites were unaffected. Despite the increased TP concentrations could be associated with greater colostrum protein content, this cannot be attributed to higher IgG absorption as no differences in circulating IgG concentrations were observed between groups. Unfortunately, no studies have reported the effects of a high‐starch diet prepartum on the transfer of passive immunity in dairy goats. Despite the present study showing an increased IgG concentration in colostrum from HS goats, no differences in plasma IgG concentrations were detected between goat kid groups. It seems that feeding high quality colostrum (i.e. ≥ 20 mg/mL of IgG; Argüello et al. 2004) might saturate the absorption capacity of immunoglobulins in the small intestine, resulting in no differences on the immunity acquired by newborn goat kids. This has been previously reported in dairy calves and goat kids fed high quality colostrum (Besser et al. 1985; González‐Cabrera et al. 2024). Indeed, Flynn et al. (2025) recently observed a lower apparent efficiency of absorption in calves fed high‐quality colostrum (24.9%) compared to those fed adequate‐quality colostrum (29.3%). In addition, some authors reported that the apparent efficiency of absorption of IgG can vary widely, ranging from 13.3% to greater than 24% in goat kids (Rodríguez et al. 2009; Geiger 2020). This high apparent efficiency of absorption variability, together with a possible selective intestinal absorption (Latvala et al. 2017; Weström et al. 2020), could explain the lack of differences in circulating IgG concentrations in goat kids. However, the precise physiological mechanisms behind these observations still require further research.

## 5 Conclusion

Feeding a prepartum high‐starch diet during the last month of gestation caused increased colostrum IgG concentration without affecting colostrum yield or chemical composition. Despite the increased colostrum IgG concentrations, prepartum diet at 32% of starch did not impact the transfer of passive immunity, metabolism and growth in the offspring. The high‐starch diet prepartum induced changes in the metabolism of the dams, reducing the extent of energy mobilisation without increasing blood glucose concentrations. Further investigation should include additional biomarkers related to energy metabolism as well as colostrum bioactive components and goat kid intestinal absorption mechanisms.

## Conflicts of Interest

The authors declare no conflicts of interest.

## Data Availability

The data that support the findings of this study are available from the corresponding author upon reasonable request.
